# A 3D Collagen–Alginate Hydrogel Model for Mechanoregulation of Autophagy in Periodontal Ligament Cells

**DOI:** 10.3390/gels12010091

**Published:** 2026-01-20

**Authors:** Xueping Kang, Bei Gao, Tong Wang, Qingbo Zhao, Shiyang Wu, Chuqi Li, Hui Zhang, Rui Zou, Yijie Wang

**Affiliations:** 1 Key Laboratory of Shaanxi Province for Craniofacial Precision Medicine Research, College of Stomatology, Xi’an Jiaotong University, Xi’an 710004, China; 2 Department of Orthodontics, College of Stomatology, Xi’an Jiaotong University, Xi’an 710004, China; 3 Bioinspired Engineering and Biomechanics Center (BEBC), Xi’an Jiaotong University, Xi’an 710049, China

**Keywords:** autophagy, periodontal ligament cells, 3D hydrogel, static compression, AKT-mTOR

## Abstract

Mechanical loading is a central cue in periodontal tissues, where compression of the periodontal ligament guides remodeling and orthodontic tooth movement (OTM). However, most mechanobiology studies have used two-dimensional cultures with poorly defined loading, and the role of autophagy under realistic three-dimensional compression remains unclear. In this study, we constructed a three-dimensional static compression model by encapsulating human periodontal ligament cells in collagen–alginate–CaSO_4_ hydrogels, whose swelling, degradation, and viscoelasticity approximate those of native matrix. When exposed to a controlled static compressive stress, the cells exhibited an early autophagic response with increased ATG7, Beclin1, and LC3-II/LC3-I; accumulation of LC3-positive puncta; and reduced p62 expression between 4 and 8 h. Pharmacological modulation showed that activation of AKT-mTOR signaling suppressed this response, whereas its inhibition further augmented autophagy, identifying AKT-mTOR as a negative regulator of compression-induced autophagy. Together, these findings demonstrate that moderate static compression drives AKT-mTOR-dependent autophagy in periodontal ligament cells and establish a simple hydrogel platform for quantitative studies of periodontal remodeling.

## 1. Introduction

Orthodontic treatment is a common clinical method for correcting malocclusions and irregular tooth alignments. Its effectiveness mainly depends on alveolar bone remodeling triggered by mechanical forces and the controlled movement of teeth. During this process, multiple cell types work together, including osteoclasts, periodontal ligament cells (PDLCs) [1,2], and immune cells [3,4,5,6]. Osteoclasts resorb bone to create space for tooth root movement [7,8], while PDLCs are responsible for new bone formation, helping to stabilize teeth in their new positions, primarily through their ability to differentiate into osteoblasts [9,10,11]. If the osteogenic function of PDLCs is impaired, patients may experience significant alveolar bone loss, tooth loosening, or even tooth loss [12]. Further research is needed to clarify how PDLCs are directed toward osteogenic differentiation during orthodontic bone remodeling.

PDLCs contribute to bone resorption by secreting inflammatory mediators and engaging in mechanical signaling under orthodontic force [8]. Recent research has identified autophagy as a critical regulator in this process. Compression can enhance autophagic activity in PDLCs on two-dimensional (2D) substrates by increasing LC3 conversion and Beclin1 expression, thereby regulating osteoclast recruitment and bone resorption during OTM [13,14,15]. However, cells within a three-dimensional (3D) matrix exhibit significant alterations in mechanical signaling and autophagic flux due to changes in integrin clustering, cytoskeletal redistribution, and nuclear deformation compared to 2D culture conditions [16,17]. The mechanisms by which 3D compression within the periodontal ligament modulates PDLCs autophagy to regulate alveolar bone resorption remain inadequately understood.

Autophagy is a conserved cellular process that maintains homeostasis under stress by directing damaged organelles and proteins to lysosomal degradation [13]. In OTM models, autophagy inhibition aggravates periodontal inflammation and accelerates alveolar bone loss, indicating that appropriate autophagic activity exerts a protective role under mechanical loading [18]. At the molecular level, mechanical cues regulate autophagy mainly through the AMP-activated protein kinase (AMPK), which promotes autophagic flux, and through the PI3K/AKT/mTOR signaling cascade, in which mTOR functions as a central suppressor of autophagosome formation [19,20,21]. Hence, we hypothesize that 3D compression modulates PDLC autophagy through the AKT-mTOR pathway to regulate bone resorption.

**Figure 1:**
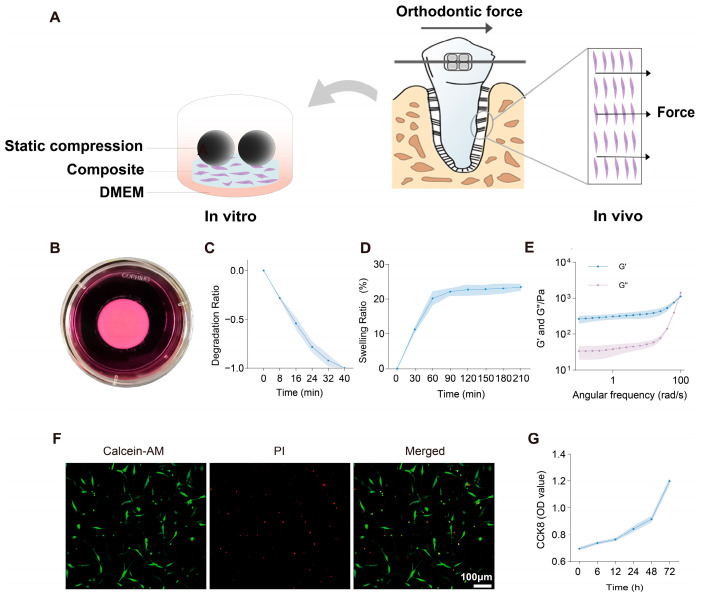
Properties of the 3D compression model for orthodontic force simulation. (**A**) Establishment and characterization of a 3D compression model mimicking orthodontic force in vitro. (**B**) Photograph of the fabricated composite hydrogel disk placed in culture medium. (**C**) Time course measurement of hydrogel degradation in enzyme solution. (**D**) Swelling ratio of composite hydrogels over the indicated time. (**E**) Rheological properties of the composite hydrogels, showing storage modulus (G′) and loss modulus (G″) across the frequency sweep. (**F**) Representative images of Calcein-AM/PI staining of encapsulated cells after culture, showing live (green) and dead (red) cells. Scale bar: 100 μm. (**G**) CCK-8 assay of cell viability within the hydrogels at different time points.

In this study, we created a 3D culture model to apply static compression. We studied the mechanism of autophagy in PDLCs and how the AKT-mTOR pathway controls this process (Figure 1A). The goal was to understand how the AKT-mTOR pathway affects autophagy under 3D compressive stress. This provides new evidence on how PDLCs are involved in tooth move during orthodontics and bone remodeling.

## 2. Results and Discussion

### 2.1. Preparation of the 3D Compression Model

Uniform composite hydrogel disks were fabricated with a thickness of 2.5 mm and a base area of approximately 2 cm^2^ (Figure 1B). Degradation behavior refers to the loss of hydrogel quality caused by enzymatic cleavage. Over time, the polymer chains in the gel are decomposed by enzymes, resulting in a decline in the quality and structural integrity of the hydrogel. Degradation was assayed in a solution containing sodium alginate hydrolysate and type IV collagenase, where the residual mass declined sharply during the first 24 min and reached complete degradation by 40 min (Figure 1C). Faster degradation can reduce the adverse effects of material residues on cells. The swelling dynamics describe the size expansion of hydrogels driven by hydration and network relaxation; that is, when the hydrogel absorbs water, the interaction between water molecules and the polymer network causes its structure to expand. Over time, the size of the hydrogel increases. Swelling measurements show a rapid increase within the first 60 min, reaching a stable value of approximately 20% of the baseline level at 90 min (Figure 1D), indicating that the hydrogel has a low swelling rate. This low swelling rate can ensure the stability of the hydrogel structure and shape, enhance the mechanical strength and elasticity of the hydrogel, and increase its stability during use. Mass changes of hydrogels measured in culture medium, reflecting the combined effects of swelling and degradation, are provided in the Supplementary Materials (Figure S1). In summary, rapid degradation and no excessive swelling help maintain the structure of the hydrogel, while ensuring that it does not interfere with the vitality and function of cells. This balance between degradation and expansion ensures that PDLCs are embedded in an environment that can simulate the native matrix of periodontal tissue.

### 2.2. Viscoelasticity and Biocompatibility of the 3D Compression Model

The viscoelastic properties of this composite hydrogel are manifested through the storage modulus (G′) and loss modulus (G″). Throughout the entire testing process, the values of the storage modulus (G′) and loss modulus (G″) remained relatively stable, and G′ was always significantly higher than G″ (Figure 1E). This indicates that the composite hydrogel has characteristics similar to those of a solid, mainly characterized by elasticity dominance. This microenvironment is closer to the condition of in vivo tissues and can provide stable mechanical support for cells. Additionally, the cell live/dead staining results showed that most cells were stained green, while only a few cells were stained red (Figure 1F), and the CCK-8 experiment results showed that cell growth was rapid after 24 h (Figure 1G). These results indicate that the PDLCs cultured in this composite hydrogel exhibit good cell vitality and proliferation ability, thereby demonstrating the excellent biocompatibility of the composite hydrogel. In summary, these mechanical properties and good biocompatibility enable this composite hydrogel to serve as an ideal platform for cell culture and mechanical biology research.

### 2.3. 3D Compression Model Promotes Autophagy of PDLCs

**Figure 2:**
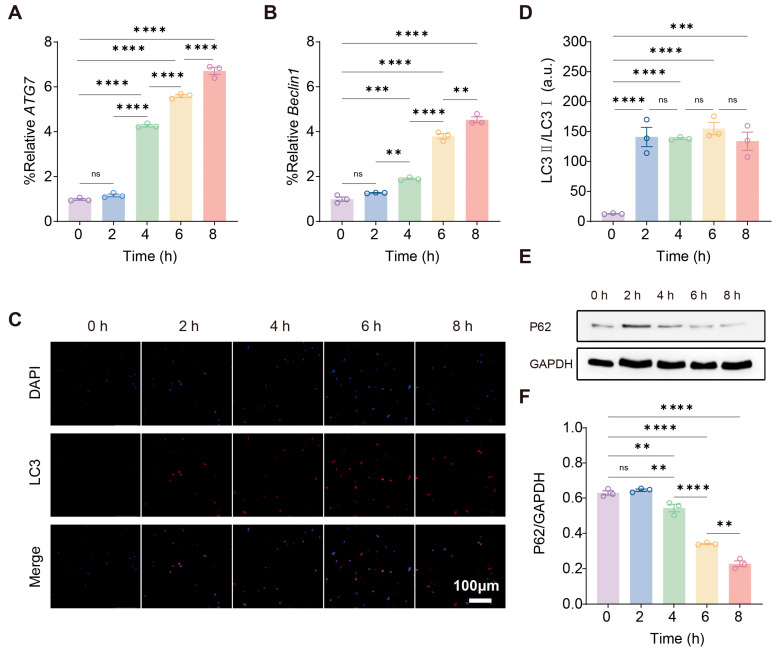
3D compression promotes autophagy of PDLCs. (**A**) qRT-PCR analysis for quantification of ATG7 expression at the indicated time points. (**B**) qRT-PCR analysis for quantification of Beclin1 expression at the indicated time points. (**C**) Representative immunofluorescence images of LC3 staining at different time points. Scale bar: 100 μm. (**D**) Ratio of LC3-II/LC3-I quantified at different time points. (**E**) Immunoblotting analysis of P62 protein levels over time. (**F**) Densitometric quantification of P62 normalized to GAPDH. For (**A**,**B**,**D**,**F**), **, ***, ****, and ns indicate *p* < 0.01, *p* < 0.001, *p* < 0.0001 and no significant difference by one-way ANOVA, respectively. Shown as the mean ± SD.

Static compression induced a time-dependent activation of autophagy in PDLCs. Autophagy-related genes showed a gradually increasing trend: ATG7 increased markedly from 4 h onward, reaching its highest level at 8 h (Figure 2A), while Beclin1 showed a similar upward trajectory with significant elevation beginning at 2–4 h and peaking at 8 h (Figure 2B). Immunofluorescence staining of LC3 demonstrated a gradual accumulation of LC3-positive puncta across the time course, with a clear enhancement of punctate fluorescence at 4–8 h (Figure 2C). Consistently, the biochemical hallmarks of autophagy activation were observed at the protein level. The LC3-II/LC3-I ratio increased progressively, with the most pronounced enhancement at later time points (Figure 2D). Studies in bone and periodontal tissues have similarly reported LC3-positive stromal cells in mechanically loaded regions and have implicated autophagy in coordinating osteoclastogenesis, extracellular matrix remodeling, and force-mediated tissue turnover [22].Concomitantly, P62 protein levels decreased in a time-dependent manner, as shown by immunoblotting (Figure 2E) and densitometric quantification (Figure 2F), indicating increased autophagic flux rather than stalled autophagosome accumulation. Collectively, these findings demonstrate that static compression triggers a time-dependent activation of the autophagy machinery in PDLCs.

### 2.4. AKT and mTOR Regulate Autophagy of PDLCs Under 3D Compression

**Figure 3:**
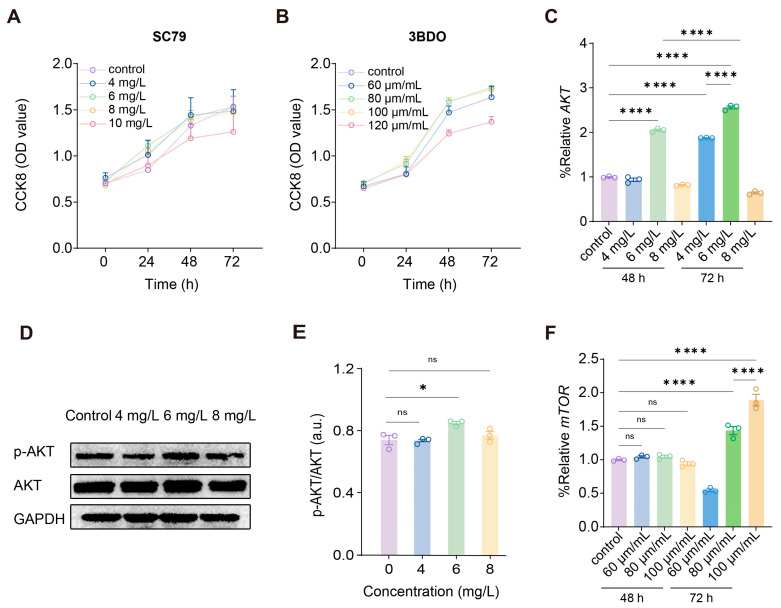
Regulation of AKT-mTOR signaling under SC79 and 3BDO treatment. (**A**) CCK8 assay of cells treated with SC79 at the indicated concentrations. (**B**) CCK8 assay of cells treated with 3BDO at the indicated concentrations. (**C**) qRT-PCR analysis for quantification of total AKT expression in response to SC79 treatment for 48 h and 72 h. (**D**) Immunoblotting analysis of p-AKT and AKT levels in cells treated with increasing concentrations of SC79. (**E**) Immunoblotting analysis for quantification of p-AKT/AKT ratios at the indicated SC79 concentrations. (**F**) qRT-PCR analysis for quantification of mTOR expression following 3BDO treatment for 48 h and 72 h. For (**C**,**E**,**F**), *, ****, and ns indicate *p* < 0.05, *p* < 0.0001 and no significant difference by one-way ANOVA, respectively. Shown as the mean ± SD.

To determine whether pharmacological modulation of AKT-mTOR signaling influences PDLCs biology under compression conditions, we first evaluated the cytotoxicity of the AKT agonist SC79 and the mTOR activator 3BDO. CCK-8 results indicated that PDLC proliferation showed no significant difference from control at SC79 concentrations of 4, 6, and 8 mg/L. In contrast, viability was significantly reduced at 10 mg/L SC79 (Figure 3A). Similarly, 3BDO at 120 μm/mL markedly inhibited PDLC growth compared with control group (Figure 3B). Therefore, we confirmed that the concentrations without inhibitory influence were SC79 at 4 mg/L, 6 mg/L, and 8 mg/L, and 3BDO at 60 μm/mL, 80 μm/mL, and 100 μm/mL in the subsequent experiments. We next assessed the activation status of AKT signaling. SC79 induced a concentration- and time-dependent increase in total AKT expression, with substantially higher levels observed at 6–8 mg/L after 72 h compared with either lower concentrations or the 48 h treatments (Figure 3C). Consistent with these findings, immunoblotting revealed enhanced phosphorylation of AKT in response to SC79 stimulation, and densitometric analysis confirmed an elevation in the p-AKT/AKT ratio, particularly at 6 mg/L (Figure 3D,E).

To investigate downstream signaling, we examined mTOR activation following 3BDO treatment. mTOR expression demonstrated a progressive elevation at 72 h, with the most prominent increase occurring at 100 μm/mL (Figure 3F). In contrast, 48 h treatments produced minimal changes, indicating that mTOR activation requires prolonged exposure to 3BDO. Overall, these data indicate that SC79 effectively augments AKT activation, whereas 3BDO preferentially enhances mTOR signaling over longer durations, and neither compound impairs cell viability across the tested dose range. These findings support a model in which mechanical compression activates autophagy at least in part through a reduction in AKT-mTOR signaling activity, consistent with the established role of this pathway as a principal negative regulator of autophagy. Similar interactions between mechanical loading and the PI3K/AKT axis have been reported in skeletal muscle [23], chondrocytes [24], and neuronal tissues [25], suggesting that force-mediated modulation of AKT signaling may represent a common mechanism across diverse cell types.

AKT is a well-established upstream regulator of the mTOR pathway, and activation of AKT is typically accompanied by downstream mTOR activation through canonical signaling cascades. Consistent with this mechanism, we observed a compression-associated reduction in p-AKT levels together with autophagy induction (Figure S2), and pharmacological modulation of AKT-mTOR signaling using SC79 and 3BDO effectively altered autophagic responses in PDLCs. These results, together with previous reports demonstrating tight coupling between AKT phosphorylation status and mTOR activity [26], support the involvement of AKT-mTOR signaling in compression-induced autophagy.

### 2.5. Pharmacological Activation of AKT-mTOR Signaling Reverses Compression-Induced Autophagy

**Figure 4:**
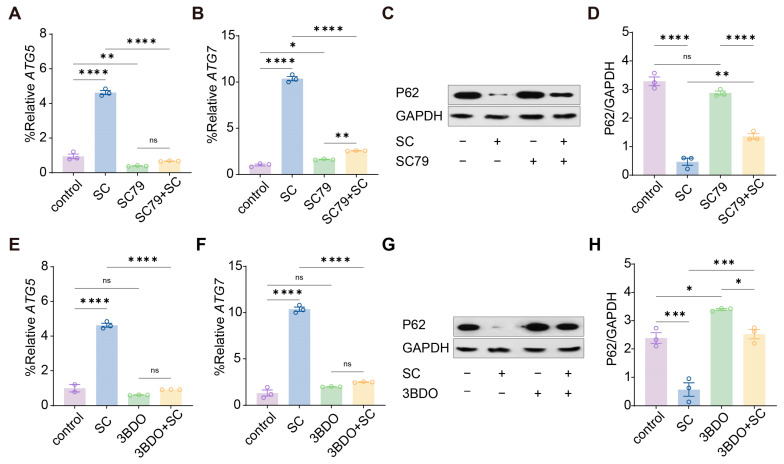
AKT mTOR activation suppresses compression-induced autophagy. (**A**) qRT-PCR analysis for quantification of ATG5 expression after SC79 and SC treatment. (**B**) qRT-PCR analysis for quantification of ATG7 expression after SC79 and SC treatment. (**C**) Immunoblotting analysis of P62 levels in cells treated with SC or SC79 alone or in combination. (**D**) Quantification of P62 relative to GAPDH corresponding to panel. (**E**) qRT-PCR analysis for quantification of ATG5 expression after 3BDO and SC treatment. (**F**) qRT-PCR analysis for quantification of ATG7 expression after 3BDO and SC treatment. (**G**) Immunoblotting analysis of P62 levels in cells treated with SC or 3BDO alone or in combination. (**H**) Densitometric quantification of P62 relative to GAPDH corresponding to panel. For (**A**,**B**,**D**–**F**,**H**), *, **, ***, ****, and ns indicate *p* < 0.05, *p* < 0.01 *p* < 0.001, *p* < 0.0001 and no significant difference by one-way ANOVA, respectively. Shown as the mean ± SD.

To investigate whether activation of AKT-mTOR signaling modifies the autophagic response caused by static compression (SC), we examined the transcriptional levels of core autophagy genes together with P62 protein abundance under SC79 or 3BDO stimulation. Compression markedly increased the expression of ATG5 and ATG7. SC79 alone produced little change, but combined treatment with SC79 and compression resulted in a pronounced reduction in both ATG5 and ATG7 toward control levels (Figure 4A,B). Consistent with the transcriptional changes, P62 protein abundance decreased under compression but was substantially restored by SC79, and cells treated with SC79 and compression showed significantly higher P62 relative to GAPDH compared with compression alone (Figure 4C,D). These findings indicate that AKT activation efficiently diminishes autophagic flux under mechanical loading.

A comparable pattern was observed for mTOR activation. Treatment with 3BDO reduced the compression induced elevation of ATG5 and ATG7 (Figure 4E,F). Immunoblot analysis showed a clear decline of P62 under compression. This reduction was partially rescued in the presence of 3BDO, and P62 relative to GAPDH increased in the 3BDO plus compression group (Figure 4G,H). Both AKT and mTOR activation consistently downregulated autophagy-related gene expression and restored P62 accumulation under compression. These results suggest that the two pathways converge to limit the autophagic response triggered by mechanical stress.

Nevertheless, the finding that autophagy remained modestly elevated even after AKT-mTOR activation suggests that additional mechanotransduction pathways contribute to this process. Candidates include MAPK/ERK signaling [27], AMPK activation [28,29], cytoskeletal tension-dependent pathways [26], and ion channel mechanosensing [30], all of which have been implicated in the regulation of force-dependent autophagy. Future studies integrating pharmacological inhibition, single-cell transcriptional analyses, and real-time imaging of autophagosome dynamics may help clarify how these pathways intersect with AKT-mTOR during force-induced remodeling.

Accumulating evidence suggests that mechanical stimulation regulates PDLCs behavior through coordinated activation of autophagy, inflammatory signaling, and bone remodeling pathways. Previous studies under 2D compression conditions have shown that mechanical stress can modulate osteogenic-related gene expression and influence osteogenic differentiation of PDLCs [11]. In the present study, we focused on the early autophagic response of PDLCs under 3D static compression, which may represent an upstream adaptive event in response to mechanical stress. Within this context, compression-induced autophagy may contribute to cytoprotective regulation of apoptosis, modulation of inflammatory cytokine secretion [31] (e.g., RANKL, TNF-α, and IL-1β), and downstream remodeling-related processes involving osteogenic [32] and osteoclastogenic [33] signaling. Together, these findings support the notion that autophagy serves as an important regulatory interface linking mechanical cues to subsequent biological behaviors during periodontal remodeling. Extending the analysis to 3D compression may provide further insight into how early autophagic responses influence subsequent biological behaviors involved in periodontal remodeling.

## 3. Conclusions

In this study, we established a 3D collagen–alginate composite hydrogel platform for mimicking static compression of the native periodontal ligament matrix. The composite hydrogels exhibited dimensional stability and favorable cytocompatibility, thereby providing a physiologically relevant 3D microenvironment for the encapsulation and maintenance of PDLCs. This platform enables reproducible delivery of controlled compressive cues that are closer to in vivo conditions than conventional two-dimensional loading systems.

**Figure 5:**
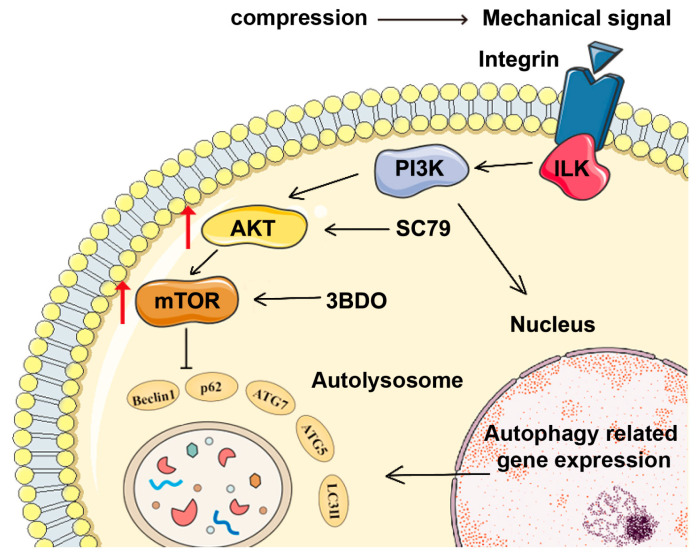
AKT-mTOR signaling links compression to autophagy in PDLCs. Compression is transmitted through AKT-mTOR signaling. Modulation of this pathway by the AKT activator SC79 and the mTOR pathway modulator 3BDO alters autophagy-related gene expression. Changes in Beclin1, P62, ATG7, ATG5 and LC3-II reflect the formation of autolysosomes and the overall level of autophagic activity in periodontal ligament cells under compression. The light red arrows indicate the increase in the levels of AKT and mTOR.

Using this system, we provide direct evidence that moderate static compression induces a rapid and time-dependent activation of autophagy in PDLCs, characterized by increased Beclin1, ATG7, and LC3-II/LC3-I expression together with decreased P62 accumulation. Pathway-targeted pharmacological modulation identifies AKT-mTOR as a major inhibitory regulator of static compression-induced autophagy in PDLCs. These findings extend previous observations obtained in 2D cultures to a well-controlled 3D context and support autophagy as an early conserved adaptation to compressive cues during orthodontic tooth movement. Collectively, this work offers a reproducible methodological model for periodontal mechanobiology research and provides functional insight into AKT-mTOR-dependent regulation of autophagy, laying a foundation for the refinement of future mechanotherapeutic strategies aimed at optimizing periodontal tissue remodeling (Figure 5).

## 4. Materials and Methods

### 4.1. Preparation of the 3D Cell Culture Model (The Type I Collagen–Alginate–CaSO_4_ Hydrogels)

The preparation of the composite hydrogels followed our previous study [34]. Briefly, type I collagen at the concentration of 1.2% (*w*/*v*), sodium alginate (Sigma-Aldrich, St. Louis, MO, USA) at 15% (*w*/*v*), 0.5 mM DGL (Alfa Aesar, Lancashire, UK), and CaSO_4_ (Kemiou, Tianjin, China) added from a 1 M solution were mixed in DMEM (Gibco, Grand Island, NY, USA). Homogeneous dispersion of all components was mixed by shaking at 37 °C prior to gelation. The pH was adjusted to neutral with NaOH (Tianli, Tianjin, China) solution. After gelation at 37 °C, the hydrogels were transferred into 35 mm wells, immersed in DMEM (Gibco) containing 15% FBS (Gibco) and 1% penicillin–streptomycin solution (Solarbio, Beijing, China), and cultured at 37 °C with 5% CO_2_. Medium was refreshed every 72 h, after which static stress was applied.

### 4.2. Swelling and Degradation Characteristics of the Composite Hydrogels

The composite hydrogels molded into a disk shape (2.5 mm × 10 mm, *n* = 6) were weighed (W_0_) and immersed in PBS at 37 °C. At each time point (30, 60, 90, 120, 150, 180, and 210 min), the hydrogels were blotted and weighed (W_1_). Swelling ratio was calculated as SR = (W_1_ − W_0_)/W_0_.

For degradation, the model disks were incubated in a 3 mL of alginate lysis buffer containing type IV collagenase at 37 °C. The initial weight (M_0_) and weights at 8, 16, 24, 32, and 40 min (M_1_) were recorded. The degradation ratio was calculated as DR = (M_1_ − M_0_)/M_0_.

### 4.3. Rheological Characteristics of the Composite Hydrogels

The rheological properties were measured using an MCR 302 rheometer (Anton Paar, Graz, Austria) with a 10 mm diameter parallel plate at 25 °C. For each test specimen, a linear region with a strain of 1% was selected for dynamic frequency scanning, and 16 tests were conducted to evaluate its storage modulus (G′) and loss modulus (G″). And loss coefficient was calculated as tanδ = G″/G′.

### 4.4. 3D Compression Model

The stress testing mold, steel balls, and cotton balls were assembled to achieve a total loading weight of 5 g. Based on our previous work [34], a compressive stress of 2.5 g/cm^2^ was continuously applied to PDLCs-encapsulated composite hydrogels for 0, 2, 4, 6, or 8 h. After loading, hydrogels were rinsed with sterile PBS and transferred to 15 mL centrifuge tubes, followed by enzymatic digestion using type IV collagenase (1 mg/mL) and alginate lyase (250 μg/mL) under gentle shaking until complete dissolution. Cell suspensions were then collected by centrifugation (1000 rpm, 5 min), washed twice with PBS, and resuspended to obtain purified cells for subsequent analyses.

### 4.5. Isolation and Identification of Human PDLCs

Periodontal ligaments were collected from orthodontic patients aged 10–14 who had their healthy premolars extracted for orthodontic reasons with informed consent and approved by the Ethics Committee of Xi’an Jiaotong University Health Science Center (Number: 2020-641). Periodontal ligaments were digested with type I collagenase (Sigma-Aldrich) and neutral enzyme (Sigma-Aldrich) for 40 min at 37 °C, then cultured in DMEM (Gibco) containing 20% FBS (Gibco) and 1% penicillin–streptomycin (Solarbio) in 5% CO_2_ at 37 °C. The third to fifth passage cells were used for subsequent experiments.

### 4.6. Cell Viability and Proliferation

A total of 5 × 10^3^ cells were encapsulated in 50 µL collagen–alginate hydrogels in a 96-well plate, and cultured in 200 µL DMEM. CCK-8 assay was performed at 0, 12, 24, 48, 72 h by adding 10 µL CCK-8 (Solarbio) and 100 µL DMEM, followed by 2 h incubation at 37 °C. Optical density (OD) values were measured at a wavelength of 450 nm using a microplate reader (Thermo Fisher Scientific, Waltham, MA, USA).

For live/dead staining, hydrogels were incubated with Calcein-AM and PI (Solarbio) for 30 min after washing, and imaged using a fluorescence microscope (Olympus, Tokyo, Japan) at 490 nm.

### 4.7. qRT-PCR

RNA was extracted using RNAzol (Invitrogen, Waltham, MA, USA), and cDNA synthesized with PrimerScript^TM^ RT Master Mix (Takara, Tokyo, Japan). Quantitative qRT-PCR was performed using the real-time fluorescent quantitative PCR system (Applied Biosystems, Foster City, CA, USA) and SYBR Premix Ex TaqTM II Kit (Takara). The primers were custom-designed from NCBI and are shown in Table 1.

### 4.8. Western Blotting Analysis

Cells were lysed in RIPA lysis buffer (Beyotime, Shanghai, China) containing protease inhibitor, and protein concentrations were determined using a BCA kit (Beyotime). Protein samples (20 μg in each group) were separated by sodium dodecyl sulfate–polyacrylamide gel electrophoresis (SDS-PAGE) and transferred to polyvinylidene fluoride (PVDF) membranes, and incubated with antibodies against GAPDH (1:5000), AKT (1:1000), p-AKT (1:1000), or P62 (1:500) (Solarbio), and then with horseradish peroxidase-conjugated secondary antibody for 1 h. Bands were visualized using an Omega Lum G imaging system (Aplegen, Pleasanton, CA, USA), and quantified using ImageJ software (version 1.53t, NIH, Bethesda, MD, USA).

### 4.9. Immunofluorescence Staining

The hydrogels were fixed with 4% paraformaldehyde, treated with 0.1% Triton X-100, and blocked with 5% BSA (Boster, Wuhan, China). Samples were incubated with LC3 primary antibody (1:200) overnight at 4 °C, followed by secondary antibody and DAPI staining (Boster). Images were captured using a confocal microscope (Olympus, Tokyo, Japan) and analyzed with ImageJ software (version 1.53t).

### 4.10. Statistical Analysis

Statistical analyses were performed using GraphPad Prism software (version 10.1.2). Statistics are presented as mean  ±  standard deviation (SD), *t*-test and one-way analysis of variance (ANOVA) were used to analyze the statistical significance of differences, with *p* < 0.05 considered statistically significant. (* *p*  <  0.05, ** *p*  <  0.01, *** *p*  <  0.001, **** *p*  <  0.0001). Each experiment was performed at least three times.

## Figures and Tables

**Table 1:** Sequences of forward and reverse primers for qRT-PCR.

Target Gene (Human)	Primer Sequences
GAPDH	Forward: ACCCACTCCTCCACCTTTG
	Reverse: CACCACCCTGTTGCTGTAG
Beclin1	Forward: GGTGTCTCTCGCAGATTCATC
	Reverse: TCAGTCTTCGGCTGAGGTTCT
ATG7	Forward: ACGGTGATGCTGTTGGTCTG
	Reverse: TTTGTCGGTGGATTTGAAGG
ATG5	Forward: GGTGTCTCTCGCAGATTCATC
	Reverse: TCAGTCTTCGGCTGAGGTTCT
AKT	Forward: GGCTCATGGTTAGATCTGGC
	Reverse: TGACCAATACTTGGTGCTTCC
mTOR	Forward: TCAAGCAGGAGTGCAATCG
	Reverse: AGAATGCCTCCTCACACAGG

## Data Availability

The original contributions presented in this study are included in the article/Supplementary Materials. Further inquiries can be directed to the corresponding authors.

## Supplementary Materials

The following supporting information can be downloaded at: https://www.mdpi.com/article/10.3390/gels12010091/s1, Figure S1: Mass change of hydrogels in DMEM during PDLCs culture. Figure S2. (A) Densitometric quantification of AKT normalized to GAPDH at the indicated SC79 concentrations. (B) Densitometric quantification of p-AKT normalized to GAPDH at the indicated SC79 concentrations.

## Abbreviations

OTM	orthodontic tooth movement
PDLCs	periodontal ligament cells
2D	two-dimensional
3D	three-dimensional
SC	static compression
AMPK	AMP-activated protein kinase
G′	storage modulus
G″	loss modulus
DGL	D-(+)-glucono-1,5-lactone
PVDF	polyvinylidene fluoride
