# Supplementary material for: A 3D Collagen–Alginate Hydrogel Model for Mechanoregulation of Autophagy in Periodontal Ligament Cells
Source: Gels. 2026 Jan 20;12(1):91. doi: 10.3390/gels12010091 (PMC12841157; doi:10.3390/gels12010091)
Supplement: Supplementary file 1 [file gels-12-00091-s001.zip › gels-4064627-supplementary.pdf]

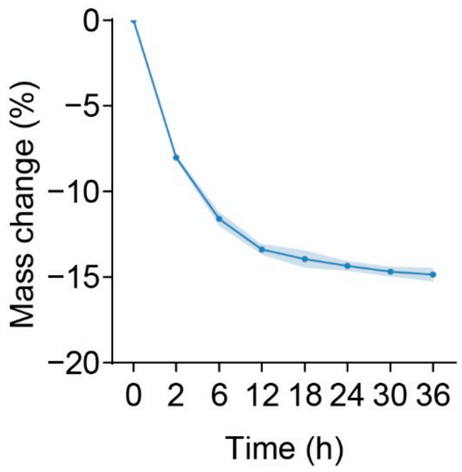

Figure S1. Mass change of hydrogels in DMEM during PDLCs culture.

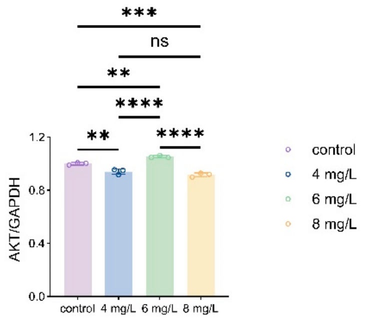

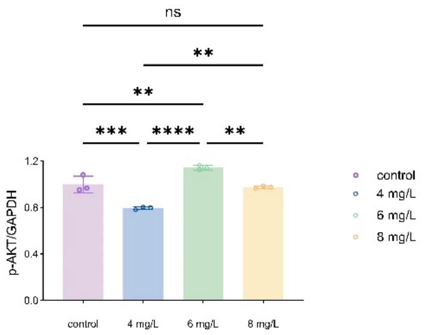

Figure S2. (A) Densitometric quantification of AKT normalized to GAPDH at the indicated SC79 concentrations. (B) Densitometric quantification of p-AKT normalized to GAPDH at the indicated SC79 concentrations.
